# Exploring the osteoporosis treatment gap after fragility hip fracture at a Tertiary University Medical Center in Thailand

**DOI:** 10.1186/s12877-023-03778-5

**Published:** 2023-02-03

**Authors:** Chantas Mahaisavariya, Ekasame Vanitcharoenkul, Nitchanant Kitcharanant, Pojchong Chotiyarnwong, Aasis Unnanuntana

**Affiliations:** 1 Golden Jubilee Medical Center, Faculty of Medicine Siriraj Hospital, Mahidol University, Bangkok, Thailand; 2 Department of Orthopaedic Surgery, Faculty of Medicine Siriraj Hospital, Mahidol University, 2 Wanglang Road, Bangkoknoi, Bangkok, 10700 Thailand; 3 Department of Orthopaedics, Faculty of Medicine, Chiang Mai University, Chiang Mai, Thailand

**Keywords:** Anti-osteoporosis medication, Hip fracture, Prescription rate, Non-persistence, Not receiving

## Abstract

**Background:**

(1) To evaluate the prescription rate of anti-osteoporosis medication, and (2) to identify factors associated with patients not receiving anti-osteoporosis medication or, when prescribed, not persisting with medication 1 year after hip fracture treatment.

**Methods:**

We retrospectively reviewed the medical records of all fragility hip fracture patients admitted to the orthopedic unit of the Faculty of Medicine Siriraj Hospital, Mahidol University, between July 1, 2016, and December 31, 2019. We identified patients who did not receive anti-osteoporosis medication both 6 months and 1 year after fracture treatment. Patients who did not receive the medication 1 year after their treatment were enrolled and interviewed using a no-treatment questionnaire.

**Results:**

In total, 530 patients with fragility hip fractures were eligible (mean age, 79.0 years), and most (74.5%) were women. Only 148 patients (31.6%) received anti-osteoporosis medication 1 year after hip fracture. Logistic regression analysis identified predictors for not receiving the medication: male sex (OR 1.8; 95% CI 1.1–3.0), Charlson comorbidity index score ≥ 5 (OR 1.5; 95% CI 1.0–2.3), and secondary school education or below (OR 2.0; 95% CI 1.2–3.3). The main reason for not receiving the medication was that healthcare providers neither discussed nor initiated pharmacological treatment for osteoporosis (48.2%). When the medication was prescribed, non-persistence primarily stemmed from transportation difficulties that resulted in patients missing follow-ups (50.0%).

**Conclusions:**

Improved physician attitudes toward anti-osteoporosis medications might enhance the treatment rate. Developing a follow-up team and facilitating access to medications (eg, courier delivery to patients) would promote therapy compliance.

**Trial registrations:**

The protocol for the first phase and second phase was approved by the Siriraj Institutional Review Board of the Faculty of Medicine Siriraj Hospital, Mahidol University, Bangkok, Thailand (COA no. Si 180/2021) and for the second phase, patients-informed consent forms used in the cross-sectional component were approved by the Siriraj Institutional Review Board of the Faculty of Medicine Siriraj Hospital, Mahidol University, Bangkok, Thailand (COA no. Si 180/2021). The research was registered with the Thai Clinical Trials Registry (TCTR number: 20210824002). The study was conducted in accordance with the Declaration of Helsinki. Each patient (or a relative/caregiver) provided informed consent in writing or by telephone to participate in this second study phase.

**Supplementary Information:**

The online version contains supplementary material available at 10.1186/s12877-023-03778-5.

## Background

With medical advances increasingly prolonging human life expectancy, the world is experiencing growth in the size and proportion of the population that is elderly [1]. The emergence of an aging society in many countries has increased the incidence of age-related diseases, including osteoporosis, accompanied by its most serious complication, fragility fracture. The annual number of hip fractures has been projected to increase from 1.66 million in 1990 to 6.26 million in 2050 [2]. Fragility fractures usually result in functional disability, morbidity, mortality, and a significant economic burden on healthcare systems [3]. For instance, the 1-year mortality rate after fragility hip fracture has been reported to be as high as 20% to 33%, with less than half of the cases returning to their pre-injury ambulatory status [4]. In addition, among those who have already sustained a fragility hip fracture, there is a high probability of recurrent falls and subsequent fractures within the next 12 to 24 months (the so-called “imminent risk of fracture”) [5]. Johansson et al. [6] found that the risk of a subsequent major osteoporotic fracture within 1 year after a fragility fracture was significantly increased, by approximately 2.7-fold, compared with that of the general population. Therefore, prescribing anti-osteoporosis treatment is critically important and should be initiated as early as possible.

Once a fragility hip fracture occurs, an anti-osteoporosis medication should be given to prevent subsequent fractures. The medication reduces the risk of subsequent vertebral and nonvertebral fractures, with reported rates of decline of 23% to 62% and 36% to 41%, respectively [7]. Although many anti-osteoporosis drugs are available, the rate of treatment with one is surprisingly low [8]. A study drawing upon data from a Canadian registry from 1996 to 2008 reported that fewer than 16% of patients who sustained a fragility hip fracture were prescribed anti-osteoporosis drugs [9]. The reasons for not receiving these medications are multifactorial and are influenced by differences in countries’ healthcare systems. A 2019 online survey conducted in Thailand by Kittithamvongs and Pongpirul [10] revealed that the main reason for not prescribing anti-osteoporosis medications was their high costs. This reason, however, was obtained solely from healthcare providers. Since the patient perspective is also critical, the reasons for not receiving these medications are still not fully elucidated.

Identifying the factors associated with not receiving anti-osteoporosis medications and with non-persistence with anti-osteoporosis medications that have been prescribed is essential. This study set out to determine the following:

- The prescription rate of anti-osteoporosis medications 1 year after fracture treatment
- Factors associated with not receiving anti-osteoporosis medications
- The causes of not receiving anti-osteoporosis medications 1 year after hip fracture treatment

## Methods

We conducted this study in 2 phases: a retrospective chart review followed by a cross-sectional analysis of the patient cohort. The protocol for the first phase and second phase was approved by the Siriraj Institutional Review Board of the Faculty of Medicine Siriraj Hospital, Mahidol University, Bangkok, Thailand (COA no. Si 180/2021) and for the second phase, patients-informed consent forms used in the cross-sectional component were approved by the Siriraj Institutional Review Board of the Faculty of Medicine Siriraj Hospital, Mahidol University, Bangkok, Thailand (COA no. Si 180/2021). The research was registered with the Thai Clinical Trials Registry (TCTR number: 20210824002). The study was conducted in accordance with the Declaration of Helsinki. Each patient (or a relative/caregiver) provided informed consent in writing or by telephone to participate in this second study phase.

### Retrospective phase

In the first phase, we retrospectively analyzed the medical records of all fragility hip fracture patients who were admitted to the orthopedic unit of the Faculty of Medicine Siriraj Hospital, Mahidol University, between July 1, 2016, and December 31, 2019. The included study population was patients diagnosed with a fragility hip fracture who were aged 50 years or older and had a minimum of 1-year follow-up data after their fracture. Patients were excluded if they had a pathological fracture (confirmed by a pathological report) or an atypical femoral fracture. We identified patients who did not receive anti-osteoporosis medication both 6 months and 1 year after fracture treatment. The data used in this study were collected from our institution’s Fracture Liaison Service Registry.

Briefly, once osteoporotic hip fractures were diagnosed, patients were admitted and received operative treatment if there were no contraindications. After the treatment, osteoporosis education was given to the patients and their family members or caregivers. Basic metabolic laboratory and radiographic investigations were performed (serum calcium, serum phosphate, renal and liver function tests, serum 25-hydroxyvitamin D, and bone mineral density testing via dual-energy X-ray absorptiometry scanning). A metabolic bone disease specialist advised the primary team of appropriate anti-osteoporosis agents based on the national osteoporosis treatment guideline and each patient’s healthcare reimbursement coverage [11]. Calcium and vitamin D supplementation was provided to all patients. Based on the 2016 Thai Osteoporosis Foundation guideline, oral bisphosphonate was the first-line medication for osteoporosis patients, including those with low-energy hip and spine fractures. Oral bisphosphonate was the only anti-osteoporosis agent registered for national use and all healthcare reimbursement systems. Parenteral anti-osteoporosis medications (intravenous bisphosphonate, denosumab, and anabolic agents) were recommended to patients who had contraindications or serious adverse events to oral bisphosphonate or who failed bisphosphonate treatment. Following hip fracture treatment, a nurse coordinator followed up with each patient 6 months, 1 year, and annually thereafter.

We collected the following demographic and clinical characteristics: age, sex, body mass index, Charlson comorbidity index (CCI) score, patient education level, prefracture ambulatory status, type of hip fracture, type of hip fracture treatment, and healthcare reimbursement coverage.

The proportion of patients who received an anti-osteoporosis agent 1 year after their hip fracture was calculated. Patients who received the medication at the 1-year timepoint were designated as the “treatment group,” and those without treatment were designated as the “no treatment group.” The baseline patient demographic and clinical characteristics of the 2 groups were compared.

### Cross-sectional phase

In the second phase, we enrolled only fragility hip fracture patients who did not receive anti-osteoporosis medication 1 year after their hip fracture treatment. We excluded patients with severe cognitive or neurological impairments that might affect their ability to respond to a questionnaire. Additionally, patients were excluded if they had chronic kidney disease stage 5 (estimated glomerular filtration rate < 15 mL/min/1.73m^2^) or end-stage renal disease (ESRD). Both conditions are contraindicated for most anti-osteoporosis medications, including our first-line anti-osteoporosis agent, oral bisphosphonate.

Each patient (or a relative/caregiver) provided informed consent in writing or by telephone to participate in this second study phase. All patient information was kept confidential. The study design and reporting format followed the Strengthening the Reporting of Observational Studies in Epidemiology (STROBE) principles. All eligible patients who met the inclusion criteria of the cross-sectional phase were interviewed using a no-treatment questionnaire. A research assistant interviewed individual patients either face-to-face or by telephone.

### No-treatment questionnaire

The “no-treatment” questionnaire used in the second phase was developed from responses to open-ended questions of a pilot group of 30 patients who did not receive any anti-osteoporosis medication 1 year after their hip fracture. Their responses were categorized into the 5 domains detailed in Supplementary Table. Briefly, the domains were as follows:

- no recommendation was made for anti-osteoporosis medication by the surgeon or primary care physician
- financial constraints (unable to afford the drug cost regardless of the patient’s healthcare reimbursement coverage)
- medication problems (experience of side effects)
- patient perception against anti-osteoporosis medications
- other reasons (eg, medical condition worsening during osteoporosis treatment, such as declining renal function)

### Statistical analyses

Since the primary outcome of this study was the prevalence of patients who did not receive anti-osteoporosis medication 1 year after their hip fracture treatment, we performed a priori sample-size calculation using data from a previous study [12]. The research reported that the prevalence of patients receiving an anti-osteoporosis agent 1 year after the fracture was 24.8%. Thus, 289 patients were determined to be needed for our study, with a confidence level of 95% and an allowable error of 5%.

Data are presented as the mean and standard deviation for continuous variables and as frequency and percentage for categorical variables. Each variable and outcome measure were assessed for normality using the Shapiro–Wilk test. In addition, Student’s unpaired *t*-test was used to compare the quantitative variables of the “treatment” and “no treatment” groups. The chi-squared test was used to compare the categorical variables of the 2 groups. The reasons for not receiving treatment 1 year after hip fracture are presented as numbers and percentages.

Following the initial analysis, a multiple logistic regression model was created to evaluate each potential explanatory factor associated with not receiving an anti-osteoporosis medication. Using forward-stepwise selection, variables that failed to achieve a probability (*p*) value of 0.20 or less were removed from the final model. A *p* value less than 0.05 was regarded as being statistically significant. All analyses were performed using PASW Statistics for Windows, version 18.0 (SPSS Inc, Chicago, IL, USA).

## Results

Between July 1, 2016, and December 31, 2019, 546 patients were enrolled in the Siriraj Fracture Liaison Service Registry. Of those, 16 patients sustained other types of fragility fractures. Therefore, 530 patients with fragility hip fractures were included in this study. The patient demographic and clinical characteristics are listed in Table 1. The mean age of the population was 79.0 years, and the majority (74.5%) were women. Over half of the patients had a CCI score of ≥ 5, and the population’s average body mass index was 22.4 kg/m^2^. Femoral neck fracture was the most common fracture type (51.7%), followed by intertrochanteric femoral fracture (46.8%) and subtrochanteric fracture (1.5%). Before the fracture, 302 patients (57.0%) could ambulate outdoors independently, and 289 patients (54.5%) did not use an assistive device.

**Table 1:** Patient demographics and clinical characteristics

Clinical variables	Total (*N* = 530)
Age (years), mean ± SD	79.0 ± 9.6
Sex; female, n (%)	395 (74.5)
Body mass index (kg/m^2^), mean ± SD	22.4 ± 5.0
Charlson comorbidity index (CCI), n (%)	
- 1–2	39 (7.4)
- 3–4	204 (38.5)
- ≥ 5	287 (54.2)
Level of education, n (%)	
- Primary school or below	352 (66.4)
- Secondary school or diploma	93 (17.5)
- Bachelor’s degree or higher	85 (16.0)
Type of hip fracture, n (%)	
- Femoral neck fracture	274 (51.7)
- Intertrochanteric fracture	248 (46.8)
- Subtrochanteric fracture	8 (1.5)
Type of treatment, n (%)	
- Conservative	45 (8.5)
- Surgical	
- Cephalomedullary nail fixation	213 (40.2)
- Bipolar hemiarthroplasty	203 (38.3)
- Dynamic hip screw fixation	32 (6.0)
- Multiple screws fixation	21 (4.0)
- Total hip arthroplasty	16 (3.0)
Pre-fracture ambulatory status, n (%)	
- Outdoor independent	302 (57.0)
- Indoor independent	153 (28.9)
- Outdoor dependent	12 (2.3)
- Indoor dependent	49 (9.2)
- Bedridden	14 (2.6)
Use of assistive device n (%)	
- No	289 (54.5)
- Yes	241 (45.5)
Healthcare reimbursement coverage, n (%)	
- Yes	377 (71.1)
- No or self-pay	153 (28.9)

**Fig. 1:**
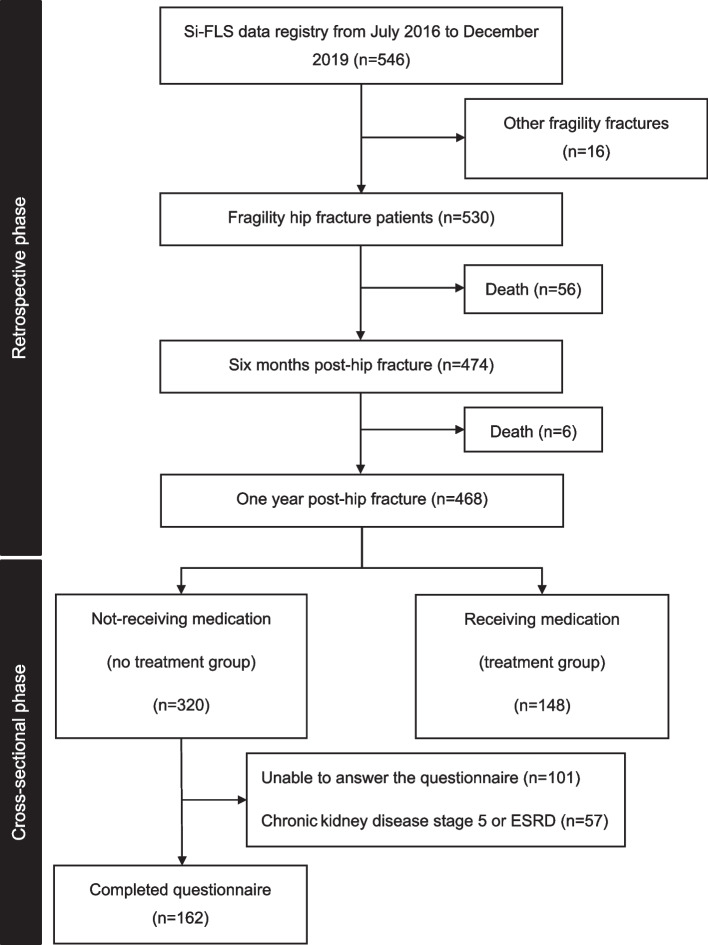
Flow diagram depicting the phases in this study (Abbreviation: Si-FLS, Siriraj Fracture Liaison Service)

During the first phase of this study, 62 patients (10.6%) died within 12 months after their hip fracture. Therefore, 468 were left for the analysis of the treatment being given 1 year after fracture treatment (Fig. 1). We found that only 148 patients (31.6%) were receiving anti-osteoporosis medication. After excluding those with advanced chronic kidney disease (stage 5) and ESRD, the number receiving anti-osteoporosis medication rose slightly to 36.0%. The most commonly prescribed anti-osteoporosis agent was oral bisphosphonate (68.5%), followed by denosumab (18.7%), intravenous bisphosphonate (8.4%), and teriparatide (4.4%).

A comparison was made of the demographic and clinical characteristics of the patients receiving and not receiving an anti-osteoporosis medication 1 year after their hip fracture. The patients who did not receive the medication were more likely to be male (*p* = 0.014), have high comorbidity (a CCI score ≥ 5; *p* = 0.027), and have a secondary school education level or lower (*p* = 0.009; Table 2). Logistic regression analysis revealed that after controlling for other risk factors, the chief predictors for not receiving anti-osteoporosis medication were (1) being male (OR 1.8; 95% CI 1.1–3.0), (2) a CCI score ≥ 5 (OR 1.5; 95% CI 1.0–2.3), and (3) a secondary school education or below (OR 2.0; 95% CI 1.2–3.3).

**Table 2:** Univariate and multivariate analyses of predictive factors of not-receiving the anti-osteoporosis medication 1 year after hip fracture

Variables	Univariate			Multivariate		
	**Treatment group** **(*n* = 148)**	**No treatment group** **(*n* = 320)**	***P*-value**	**Odds** **ratio**	**95% confidence interval**	***P*-value**
Age (years)	77.8 ± 9.1	78.9 ± 10.0	0.272			
Sex; male, n (%)	25 (16.9)	88 (27.5)	**0.014**	1.8	1.1 – 3.0	**0.017**
Body mass index (kg/m^2^)	22.1 ± 4.0	22.6 ± 4.4	0.246			
Charlson comorbidity index, n (%)						
- 1–4	82 (55.4)	142 (44.4)				
- ≥ 5	66 (44.6)	178 (55.6)	**0.027**	1.5	1.0 – 2.3	**0.045**
Level of education, n (%)						
- Secondary school or below	113 (76.4)	276 (86.2)	**0.009**	2.0	1.2 – 3.3	**0.007**
- Bachelor’s degree or higher	35 (23.6)	44 (13.8)				
Type of hip fracture, n (%)						
- Femoral neck fracture	76 (51.4)	169 (52.8)				
- Intertrochanteric fracture	68 (45.9)	147 (45.9)	0.974			
- Subtrochanteric fracture	4 (2.7)	4 (1.3)	0.267			
Pre-fracture ambulatory status, n (%)						
- Bedridden	3 (2.0)	10 (3.1)				
- Indoor dependent	10 (6.8)	28 (8.8)	0.817			
- Outdoor dependent	2 (1.4)	9 (2.8)	0.769			
- Indoor independent	45 (30.4)	86 (26.9)	0.445			
- Outdoor independent	88 (59.5)	187 (58.4)	0.502			
Use of assistive device, n (%)						
- No	81 (54.7)	177 (55.3)	0.906			
- Yes	67 (45.3)	143 (44.7)				
Healthcare reimbursement coverage, n (%)						
- Yes	113 (76.4)	240 (75.0)	0.752			
- No or self-pay	35 (23.6)	80 (25.0)				

Regarding the 320 patients who did not receive anti-osteoporosis medication 1 year after their hip fracture, 101 patients could not respond to the no-treatment questionnaire because of cognitive or neurological impairments. Another 57 patients had advanced chronic kidney disease (stage 5) or ESRD, which are contraindicated for most anti-osteoporosis agents. Thus, 162 patients (50.6%) completed the no-treatment survey. Of those, 112 had never received any anti-osteoporosis agent. The remaining 50 patients received an anti-osteoporosis agent but discontinued its use within 12 months of their fracture treatment. This latter group of patients was considered non-persistent with osteoporosis treatment. The primary reasons for not receiving any anti-osteoporosis agents (Table 3) were as follows:

- healthcare providers neither discussed nor initiated pharmacological treatment for osteoporosis (48.2%)
- financial constraints (26.8%)
- patient perception against anti-osteoporosis medication (11.6%)
- concerns about the medication’s adverse effects (8.0%)
- inappropriate medical condition (5.4%)

**Table 3:** Reasons for not receiving and non-persistence with anti-osteoporosis medication 1 year after fragility hip fracture

**Reasons for not receiving anti-osteoporosis medication 1 year after hip fracture treatment**	**Total (*N* = 112)** **n (%)**
No recommendation from surgeon and primary care physician	54 (48.2%)
Financial constraints	30 (26.8%)
Patient perception against anti-osteoporosis medication	13 (11.6%)
Concerns about adverse medication effects	9 (8.0%)
Inappropriate medical condition	6 (5.4%)
**Reasons for non-persistence with anti-osteoporosis medication 1 year after hip fracture treatment**	**Total (*N* = 50)** **n (%)**
Difficulties continuing to receive the medication	25 (50.0%)
Patient perception against anti-osteoporosis medication	12 (24.0%)
Surgeon or primary care physician discontinued the treatment without any specific reason	8 (16.0%)
Inappropriate medical condition or experienced adverse medication effects	5 (10.0%)

For the 50 non-persistent patients, the main reason for ceasing anti-osteoporosis therapy was related to transportation difficulties faced by the patients, leading to their missing follow-ups (50.0%). Other reasons for non-persistence were patient perception against anti-osteoporosis medications (24.0%) and the discontinuation of anti-osteoporosis therapy by a healthcare provider without a clear explanation given to the patient (16.0%). Only 5 patients (10.0%) had an inappropriate medical condition or experienced adverse events related to the medication, leading to its discontinuation (Table 3).

## Discussion

Patients who have sustained a recent fragility hip fracture have a very high risk of a recurrent fracture. The incidence of subsequent fracture has been reported to be 7.1% within 1 year and 12% within 2 years after the initial fracture [5]. Therefore, early initiation of anti-osteoporosis medications is of prime importance for subsequent fracture reduction. However, the global anti-osteoporosis treatment rate is surprisingly low, ranging from 22% to 40.3% [13–16]. Here, we report an anti-osteoporosis administration rate of 31.6%. The proportion rose slightly to 36.0% after excluding patients with advanced chronic kidney diseases (stage 5) and ESRD within the first year following hip fracture treatment.

The factors associated with not receiving anti-osteoporosis medication are multifactorial. Among them are national treatment guidelines, healthcare reimbursement schemes, healthcare systems, patients and caregivers’ perceptions of osteoporosis treatment, and physicians and policy makers’ beliefs about the benefits of secondary fracture prevention. For instance, Shah et al. [14] reported that the independent predictors for the non-prescription of anti-osteoporosis medications after a fragility hip fracture in the United Kingdom were being male, an increase in body mass index, and geographic region. Another study from Singapore between 2014 and 2016 found 2 predictive factors for the non-prescription of anti-osteoporosis medications 1 year after hip fracture treatment. They were being male sex and not receiving an osteoporosis investigation (bone mineral density or 25-hydroxyvitamin D level testing) after hip fracture treatment [16].

In this study, we identified that being male (OR 1.8; 95% CI 1.1–3.0), high comorbidity (CCI score ≥ 5; OR 1.5; 95% CI 1.0–2.3), and education level (secondary school or below; OR 2.0; 95% CI 1.2–3.3) were the main independent predictors for not receiving anti-osteoporosis medications. Similar to our results, other studies have reported that being male was a predictive factor for undertreatment [14, 16]. A mistaken belief that osteoporosis is a woman’s disease can explain this finding, leading to some healthcare systems not permitting the reimbursement of treatment costs for men. High CCI scores are associated with multiple coexisting diseases, complex clinical management, and increased costs resulting from treating underlying diseases. Healthcare providers’ decision-making on whether to provide anti-osteoporosis therapy might be influenced by such factors. The magnitude of the factors may also lead to the need to assess and treat osteoporosis being given low or no priority. Another risk factor is that patients with low education levels were less likely to use anti -osteoporosis medication than those with high education levels. A lack of knowledge or understanding of osteoporosis, or a misconception that anti-osteoporosis medications cannot prevent further fragility fractures, might be barriers to patients with lower levels of education accepting or persisting with therapy [17].

Understanding why patients do not receive medication is crucial to improving the overall rate of osteoporosis treatment. The reported reasons why patients declined or did not persist with the medications include a preference for alternative treatments, financial constraints, concerns about side effects, and a lack of belief in the effectiveness of the medications in preventing further fractures [18–20]. In contrast, our study revealed that nearly half of the patients stated that their surgeons or physicians did not refer to or recommend anti-osteoporosis medications. Most hip fracture patients were possibly treated by orthopedic surgeons who tended to focus on fracture treatment more than osteoporosis treatment. Another possible barrier was a lack of physician awareness of the essential need for an anti-osteoporosis medication, despite the indication for treatment. Adequate knowledge of current practice guidelines and confidence in treatment outcomes might positively influence the treatment initiation rate. Other reasons for not receiving the medication were consistent with previous studies [18–21]. Regarding non-persistence, the most common reason was transportation difficulties impeding receiving medications. This might be corrected by developing a telemedicine program and implementing a program to send patients’ medication directly to their homes to support older people and their caregivers.

A strength of the present work is that it is one of a few studies exploring why hip fracture patients do not receive medications. However, it has some mentionable limitations. First, this study collected data from only one center in Thailand, a high-volume hospital with an experienced fracture liaison service team. Consequently, some aspects of our data and findings may not be generalizable to centers that provide a less sophisticated level of care or do not have a fracture liaison service. Second, most patients had high comorbidity, indicated by their high mean CCI score. The comorbidities could have impacted the patients’ mobility and heightened their transportation difficulties more than the general older population. Third, we did not perform a subgroup analysis of the rate of treatment among the different groups of physicians, such as metabolic bone disease specialists versus nonmetabolic bone disease specialists. Such an analysis might have revealed significant differences. Finally, of those 320 patients who did not receive anti-osteoporosis medication 1 year after their hip fracture, approximately 30% did not respond to the no-treatment questionnaire. Thus, the results of this study might have changed if all patients who did not receive medication had responded. In addition, the questionnaire allowed each patient to select only the most important reason for not receiving or not persisting with medication. Since some patients might have had more than 1 reason for not receiving anti-osteoporosis medication, the various proportions of the reasons for not receiving medication 1 year after hip fracture treatment might have differed. Nevertheless, the main reason for not receiving anti-osteoporosis medication and the study conclusions would not have been affected.

## Conclusions

Despite establishing the hospital’s Fracture Liaison Service, the anti-osteoporosis treatment rate after fragility hip fracture was still low (31.6%). Male sex, high comorbidity (a CCI score ≥ 5), and secondary school education or below were strong predictive factors for not receiving anti-osteoporosis medications. The lack of a physician’s recommendation for anti-osteoporosis treatment was the primary reason given by patients who did not receive the drug. This finding underscores that improving physicians’ attitudes toward anti-osteoporosis medications might improve the rate of treatment. Transportation difficulties impeding follow-up visits were the main reason for non-persistence with treatment. Developing a follow-up team and facilitating access to medications (e.g., courier delivery to patients) would help to decrease the number of patients who discontinue anti-osteoporosis treatment.

## Supplementary Information

**Additional file 1: Supplementary Table.** Reasons for not receiving anti-osteoporotic medication.

## Data Availability

The datasets generated and/or analyzed during the current study are not publicly available due to limitations of ethical approval involving the patient data but are available from the corresponding author on reasonable request.

## Abbreviations

*CCI*: Charlson comorbidity index
*ESRD*: End-stage renal disease
