# Supplementary material for: Exploring the osteoporosis treatment gap after fragility hip fracture at a Tertiary University Medical Center in Thailand
Source: BMC Geriatr. 2023 Feb 3;23:70. doi: 10.1186/s12877-023-03778-5 (PMC9898992; doi:10.1186/s12877-023-03778-5)
Supplement: Supplementary file 1 — Additional file 1: Supplementary Table. Reasons for not receiving anti-osteoporotic medication. [file 12877_2023_3778_MOESM1_ESM.docx]

**Supplementary Table** Reasons for not receiving anti-osteoporotic medication

| **Domain** | **Description** |
| --- | --- |
| No recommendation from surgeon and primary care physician | There is no mention of the role of anti-osteoporosis agents by the patient’s orthopedic surgeon, primary physician, or other healthcare providers |
|  | Surgeon or primary care physician discontinued the treatment without any stated reason |
| Financial constraints | Unable to afford the cost of anti-osteoporosis medication regardless of patient’s healthcare reimbursement coverage |
| Drug-related problems | Experienced medication side effects |
| Patient perception against anti-osteoporosis medication | Patient’s beliefs or perception against osteoporosis treatment |
|  | Fear of medication side effects |
| Other reasons | For example, difficulties continuing to receive the medication, inappropriate medical condition, or other medical conditions that are of greater concern |
